# Complete Genome Sequences of Four African Horse Sickness Virus Strains from a Commercial Tetravalent Live Attenuated Vaccine

**DOI:** 10.1128/genomeA.01375-15

**Published:** 2015-11-25

**Authors:** Alan J. Guthrie, Peter Coetzee, Darren P. Martin, Carina W. Lourens, Estelle H. Venter, Camilla T. Weyer, Christopher Joone, Misha le Grange, Cindy K. Harper, Peter G. Howell, N. James MacLachlan

**Affiliations:** a Equine Research Centre, Faculty of Veterinary Science, University of Pretoria, Onderstepoort, South Africa; b Department of Veterinary Tropical Diseases, Faculty of Veterinary Science, University of Pretoria, Onderstepoort, South Africa; c Department of Integrative Biomedical Sciences, Computational Biology Group, Institute of Infectious Disease and Molecular Medicine, University of Cape Town, Rosebank, Cape Town, South Africa; d Veterinary Genetics Laboratory, Faculty of Veterinary Science, University of Pretoria, Onderstepoort, South Africa; e Equine Viral Diseases Laboratory, Department of Pathology, Microbiology and Immunology, School of Veterinary Medicine, University of California, Davis, California, USA

## Abstract

This is a report of the complete genome sequences of plaque-selected isolates of each of the four virus strains included in a South African commercial tetravalent African horse sickness attenuated live virus vaccine.

## GENOME ANNOUNCEMENT

African horse sickness (AHS) is an arthropod-borne equine disease caused by African horse sickness virus (AHSV) (genus *Orbivirus*, family *Reoviridae*). The AHSV genome consists of 10 segments of double-stranded RNA (dsRNA) collectively encoding seven structural proteins and four nonstructural proteins (1). We report here the genome sequences of AHSV-2/Labstr/ZAF/1998/OBP-252.1 (serotype 2), AHSV-6/Labstr/ZAF/1998/OBP-252.1 (serotype 6), AHSV-7/Labstr/ZAF/1998/OBP-252.1 (serotype 7), and AHSV-8/Labstr/ZAF/1998/OBP-252.1 (serotype 8), which were isolated from bottle II of the South African polyvalent AHS attenuated live virus (ALV) vaccine (2) (Onderstepoort Biological Products [OBP] Ltd., Onderstepoort, South Africa).

Individual serotypes were independently isolated using plaque selection on Vero cells in the presence of heterologous antibody to the other serotypes contained in the tetravalent vaccine, as previously described (3). Each of these viruses was then passaged on monolayers of baby hamster kidney (BHK-21) cells, and AHSV dsRNA was extracted, cDNA was prepared (4), and amplicons were sequenced on an Illumina MiSeq sequencer using the Nextera XT DNA sample preparation kit and 300-bp paired-end V3 Illumina chemistry, as previously described (5). Sequence reads were analyzed using *de novo* assembly, followed by mapping in Geneious 8.1.7 to obtain full-length genome sequences of the four viruses.

The genome sequences of AHSV serotype 2 (HS82/61) and serotype 6 (HS02/75) viruses, which are precursors of the respective AHS-ALV virus strains, are available from GenBank (KF859996 to KF860005 and KP009741 to KP009750, respectively). The pairwise nucleotide sequence identity of AHSV-2/Labstr/ZAF/1998/OBP-252.1 and HS82/61 was 99.536%, while that of AHSV-6/Labstr/ZAF/1998/OBP-252.1 and HS02/75 was 99.350%.

The genome sequences of the AHSV serotype 7 strain with a truncated VP2 protein (AHSV7-tVP2) (6) from which AHSV-7/Labstr/ZAF/1998/OBP-252.1 was derived are available from GenBank (accession numbers JQ742006 to JQ742015). The pairwise nucleotide sequence identity between AHSV-7/Labstr/ZAF/1998/OBP-252.1 and AHSV7-tVP2 was >99.3% for all segments, except those encoding the VP5 and nonstructural 2 (NS2) proteins, which were lower, at 77.4% and 97.9%, respectively. Analysis using RDP4.56 (7), as described previously (5), showed that AHSV-7/Labstr/ZAF/1998/OBP-252.1 is a reassortant (*P* = 7.56 × 10^-156^) composed of eight segments likely derived from AHSV7-tVP2 (accession numbers JQ742006 to JQ742010, JQ742012, JQ742013, and JQ742015), and the other segments likely derived from HS39/63 (accession numbers KF860011 and KF860013).

The genome sequences of the AHSV serotype 8 (HS10/62) from which AHSV-8/Labstr/ZAF/1998/OBP-252.1 was derived are available from GenBank (accession numbers KF860026 to KF860035). Pairwise nucleotide sequence identity between AHSV-8/Labstr/ZAF/1998/OBP-252.1 and HS10/62 over four of the 10 segments was >99.9%, while that for the remaining segments was between 76.8% and 97.4%. Analysis using RDP4.56 (7) showed that AHSV-8/Labstr/ZAF/1998/OBP-252.1 is a reassortant composed of four genome segments (accession numbers KF860026 to KF860028 and KF860034) likely derived from HS10/62, segments encoding NS1, VP5, VP7, and VP6 (accession numbers KM886348 to KM886350 and KM886352) likely derived from HS30/62 (*P* = 2.71 × 10^-282^), and the segments encoding VP4 and NS3 likely derived from an AHSV for which whole-genome sequence data are currently not available (*P* = 3.72 × 10^-126^ and 4.05 × 10^-59^, respectively).

### Nucleotide sequence accession numbers.

The AHSV-2/Labstr/ZAF/1998/OBP-252.1, AHSV-6/Labstr/ZAF/1998/OBP-252.1, AHSV-7/Labstr/ZAF/1998/OBP-252.1, and AHSV-8/Labstr/ZAF/1998/OBP-252.1 sequences have been deposited in GenBank under accession numbers KT715601 to KT715610, KT715611 to KT715620, KT715621 to KT715630, and KT715631 to KT715640, respectively.

## References

[1] Zwart L, Potgieter CA, Clift SJ, Van Staden V 2015 Characterising non-structural protein NS4 of African horse sickness virus. PLoS One 10:e0124281. doi:10.1371/journal.pone.0124281. 25915516 PMC4411093

[2] Von Teichman BF, Dungu B, Smit TK 2010 *In vivo* cross-protection to African horse sickness serotypes 5 and 9 after vaccination with serotypes 8 and 6. Vaccine 28:6505–6517. doi:10.1016/j.vaccine.2010.06.105. 20638456

[3] Howell PG, Kümm NA, Botha MJ 1970 The application of improved techniques to the identification of strains of bluetongue virus. Onderstepoort J Vet Res 37:59–66. 4340799

[4] Potgieter AC, Page NA, Liebenberg J, Wright IM, Landt O, van Dijk AA 2009 Improved strategies for sequence-independent amplification and sequencing of viral double-stranded RNA genomes. J Gen Virol 90:1423–1432. doi:10.1099/vir.0.009381-0. 19264638

[5] Guthrie AJ, Coetzee P, Martin DP, Lourens CW, Venter EH, Weyer CT, Joone C, le Grange M, Harper CK, Howell PG, MacLachlan NJ 2015 Complete genome sequences of the three African horse sickness virus strains from a commercial trivalent live attenuated vaccine. Genome Announc 3(4):e00814-15. doi:10.1128/genomeA.00814-15. 26294618 PMC4543496

[6] Manole V, Laurinmäki P, Van Wyngaardt W, Potgieter CA, Wright IM, Venter GJ, van Dijk AA, Sewell BT, Butcher SJ 2012 Structural insight into African horsesickness virus infection. J Virol 86:7858–7866. doi:10.1128/JVI.00517-12. 22593166 PMC3421665

[7] Martin DP, Murrell B, Golden M, Khoosal A, Muhire B 2015 RDP4: detection and analysis of recombination patterns in virus genomes. Virol Evol 1:vev003. doi:10.1093/ve/vev003. PMC5014473 27774277

